# Diagnosis, Prognosis and Treatment of Shock

**Published:** 1919-01

**Authors:** 


**Contributions to the Researches on the Diagnosis, Prognosis and Treatment of Shock.** By MM. Moreau and Benhamou. *La Presse Médicale*, September 26, 1918.

The authors submit all patients suspected to be suffering from shock to a careful examination with the Pachon apparatus. Three results of this examination are chiefly to be considered.

(1) The value of the *differential pressure*; i. e., the difference between the maxima (Mx) and minima (Mn) of the arterial pressure.

(2) The value of the *oscillometric index;* i. e., the greatest amplitude obtained during the examination in the oscillations of the manometer.

(3) The equality or inequality of the oscillations for a certain indication of the manometer.

The authors have determined that a failure of both the vasomotor system, and the cardiac motor itself, whatever is the cause of this failure, accompanies all cases of shock.

Every severely wounded patient, presenting a differential pressure inferior to 2, an oscillometric index lower than 1, and unequal oscillations, should be considered and treated as a shocked patient, even if his diastolic pressure is higher than 6.

The condition of a shocked patient presenting a differential pressure ranging between +2 and 5, and increasing gradually, a satisfactory oscillometric index (11/2 to 3) and even oscillations, may be considered as improving, even if, in his case, Mn is lower than 6.

The reverse of the above propositions prove, on the contrary, an aggravation in the patient's condition, even if Mn's value is higher than 6.

These conclusions are applicable only to patients wounded in the limbs, the abdomen, or the chest. Wounds in the head produce necessarily high differential pressures and oscillometric index, death in such cases occurring because of cardiac collapse.

Such being the diagnosis and prognosis in cases of shock, the authors consider that the following treatment should be given, according to the different cases:

(1) *Cases with satisfactory differential pressure and oscillometric index, and even oscillations.*

Such cases need not be dealt with as cases of urgency; they can await their turn to be carried into the operating theater. They can simply be given a tonic treatment: subcutaneous or intravenous injections of normal saline, Locke's serum, or adrenalin diluted with serum, and careful warming up.

(2) *Cases with a differential pressure equal to zero.*

Such cases seem to get no benefit from the ordinary surgical or medical treatment. What they most need is the reawakening of the cardiac contractibility; this may be obtained by means of intravenous injections of substances able to excite the contractions of the myocardium (injections of camphorated oil for instance).

If the case is one of true hemorrhage, a transfusion of blood may prove most effective.

(3) *Cases presenting a continuous and rapid fall of Mn.*

The condition of shock is generally maintained and aggravated in such cases, by some persistent hemorrhage. They need immediate operation. The cause of hemorrhage must be searched for and removed immediately. After the intervention, blood transfusion, or intravenous injections of massive doses of serum may have good results.

(4) *Cases presenting a gradual fall in the differential pressure.*

In such cases the condition of shock is occasioned, maintained, and aggravated by some poisoning or infection of the whole organism. They also need immediate operation. The lower the differential pressure falls beneath 2, and the lower the oscillometric index falls beneath 1, the more doubtful are the good results that may be expected from the intervention.

